# Don’t Bypass When There’s Bilevel Positive Airway Pressure (BiPAP): Successful Management of Respiratory Distress and Atrial Flutter in a 77-Year-Old Woman Using BiPAP

**DOI:** 10.7759/cureus.87417

**Published:** 2025-07-07

**Authors:** Angel U Davila-Cardona, Belissa A Lopez Pena, Ivan R Figueroa-Baez, Natalia Canevaro-Lugo, Juan A Ruiz Ramos

**Affiliations:** 1 Internal Medicine, San Juan City Hospital, San Juan, PRI

**Keywords:** acute decompensated heart failure, atrial flutter, autonomic dysregulation, bipap noninvasive ventilation, emergency management, hemodynamic stabilization, pulmonary edema, rapid ventricular response, sinus rhythm conversion

## Abstract

Acute decompensated heart failure (ADHF) often presents with respiratory distress and tachyarrhythmias such as atrial flutter, driven by autonomic dysregulation. Noninvasive ventilation, particularly bilevel positive airway pressure (BiPAP), can improve hemodynamic status and reduce respiratory effort. A 77-year-old woman with no prior cardiac history presented with acute shortness of breath, orthopnea, and paroxysmal nocturnal dyspnea. Examination of the ECG revealed atrial flutter with rapid ventricular response (HR: 144 bpm), hypoxemia (SpO₂: 89%), and pulmonary congestion. Imaging showed cardiomegaly and vascular redistribution; brain natriuretic peptide was elevated. She was initiated on BiPAP, resulting in rapid clinical improvement, including rate control and resolution of hypoxemia. This case demonstrates BiPAP’s hemodynamic and autonomic benefits in ADHF. Evidence supports its role in reducing sympathetic overactivity, improving heart rate variability, and decreasing preload and afterload. Early BiPAP use may prevent intubation and improve outcomes in appropriately selected patients. Early initiation of BiPAP should be considered a cornerstone in the guideline-directed management of ADHF, particularly in patients with respiratory distress and atrial flutter.

## Introduction

Acute decompensated heart failure (ADHF) is a life-threatening clinical syndrome characterized by elevated left ventricular filling pressures, pulmonary congestion, and heightened sympathetic nervous system activity, often resulting in autonomic imbalance and end-organ dysfunction [1]. Among the tachyarrhythmias seen in this setting, atrial flutter with rapid ventricular response (RVR) is frequently encountered. It exacerbates hemodynamic compromise by increasing myocardial oxygen demand, impairing diastolic filling, and reducing cardiac output [2,3].

Noninvasive ventilation (NIV), particularly bilevel positive airway pressure (BiPAP), has emerged as a cornerstone in the acute management of ADHF with respiratory distress. The 2021 European Society of Cardiology guidelines recommend early use of NIV to relieve dyspnea, reduce preload and afterload, and enhance gas exchange and hemodynamic stability [4,5]. BiPAP has also been shown to positively influence autonomic tone and reduce the work of breathing, which may in turn modulate arrhythmogenic triggers [6-8].

This case reports the clinical utility of early BiPAP intervention in an elderly patient with previously undiagnosed ADHF and atrial flutter with RVR. The prompt application of NIV led to rapid improvement in respiratory status and restoration of hemodynamic balance, emphasizing the therapeutic synergy between respiratory support and rhythm control in this population.

## Case presentation

A 77-year-old female smoker with no known medical history presented to the emergency department with a four-day history of worsening shortness of breath, orthopnea, and paroxysmal nocturnal dyspnea. Her family also noted decreased oral intake, fatigue, and increasing leg swelling. She denied fever, chest pain, or recent travel.

**Figure 1:**
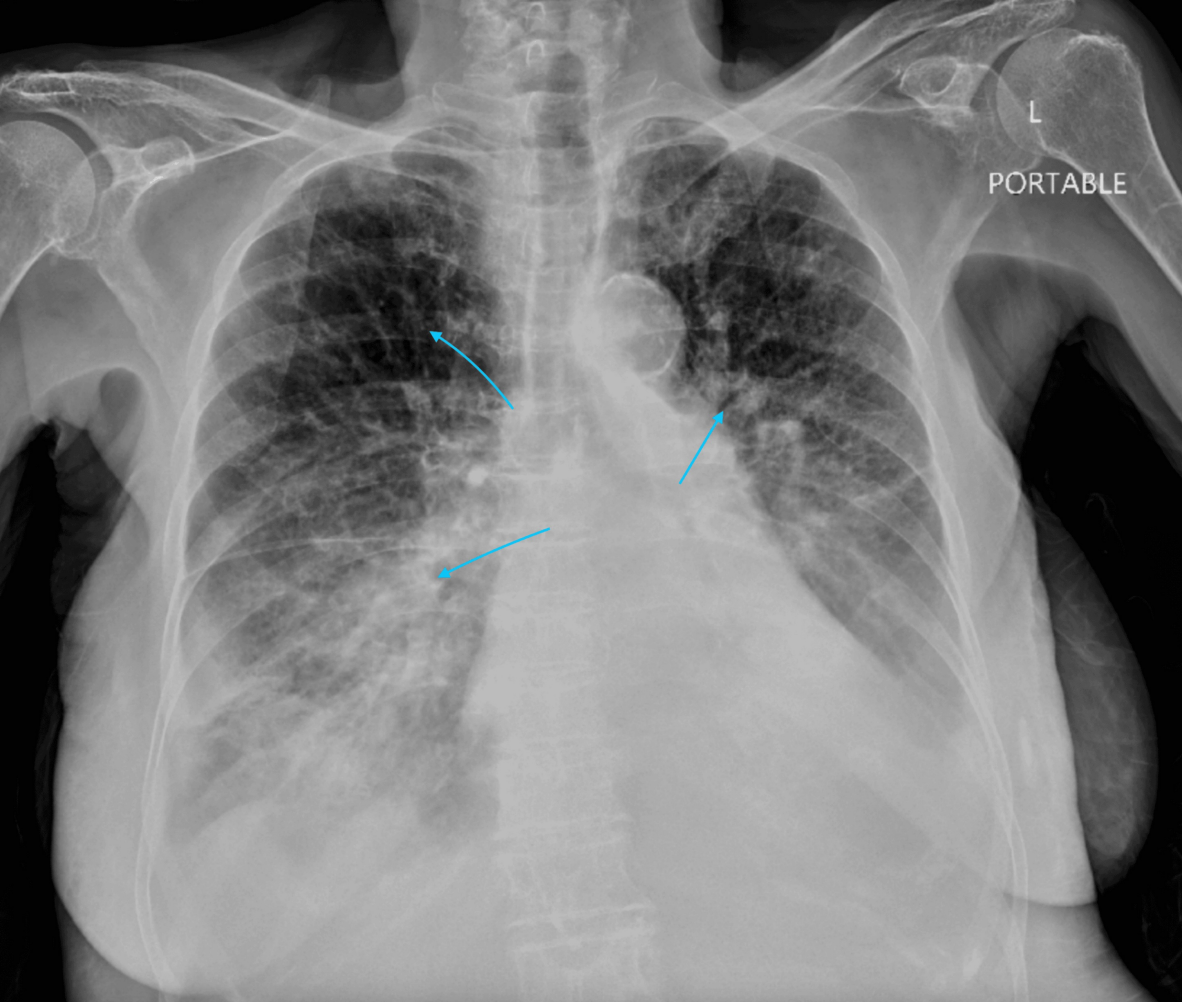
Portable chest X-ray demonstrating pulmonary vascular cephalization, bilateral perihilar congestion, and diffuse interstitial and alveolar opacities consistent with pulmonary edema in the setting of decompensated congestive heart failure

**Figure 2:**
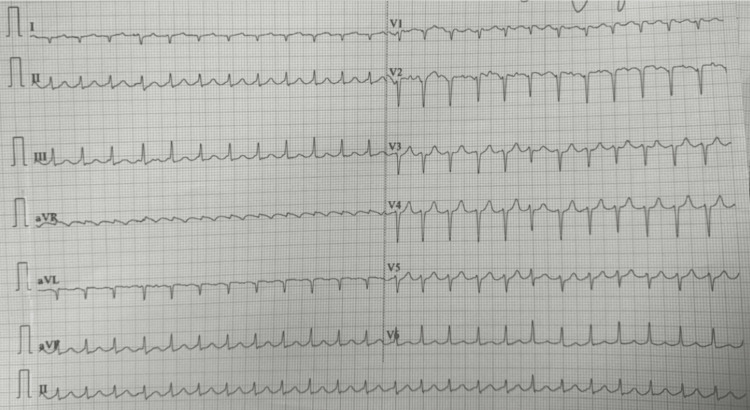
Baseline EKG on admission demonstrating atrial flutter with RVR A 12-lead EKG on arrival shows atrial flutter with 2:1 atrioventricular conduction and a ventricular rate of 144 bpm. RVR, rapid ventricular response

On examination, she appeared in respiratory distress. Vital signs were notable for a blood pressure of 160/90 mmHg, a heart rate of 164 beats per minute in atrial flutter with RVR, a respiratory rate of 28 breaths per minute, and an oxygen saturation of 89% on room air. Jugular venous distention was present, and cardiac auscultation revealed an irregularly irregular rhythm with an S3 gallop. Bibasilar crackles were heard up to the mid-lung fields. Bilateral lower extremity pitting edema was noted. Chest radiography demonstrated cardiomegaly and pulmonary vascular congestion (Figure 1). ECG confirmed atrial flutter with 2:1 AV conduction (Figure 2). Her serum BNP was 1399 pg/mL. Arterial blood gas revealed a mild respiratory alkalosis.

**Figure 3:**
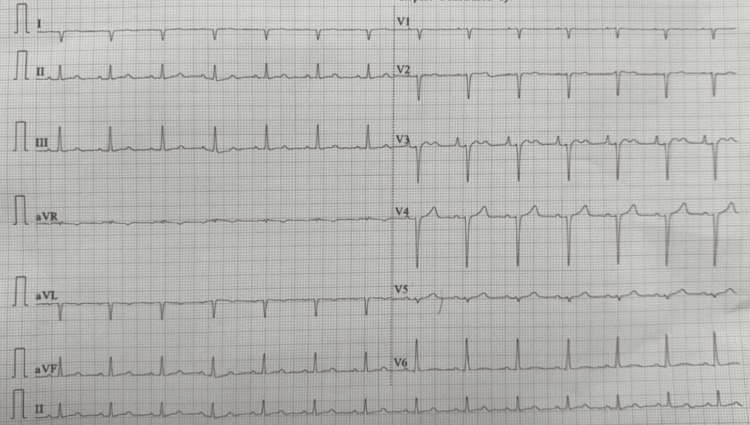
EKG post-BiPAP initiation demonstrating rate control and sinus rhythm Follow-up EKG obtained approximately 30 minutes after BiPAP initiation shows conversion to sinus rhythm with a ventricular rate of 83 bpm. BiPAP, bilevel positive airway pressure

The patient was promptly initiated on BiPAP therapy with inspiratory positive airway pressure (IPAP) of 12 cm H₂O, expiratory positive airway pressure (EPAP) of 6 cm H₂O, respiratory rate set at 20 breaths per minute, and FiO₂ of 40%. Within 30 minutes of therapy, her respiratory rate improved, SpO₂ rose to 95%, and her heart rate decreased to 83 bpm (Figure 3). She was also treated with intravenous furosemide and high-dose nitroglycerin for afterload reduction. Echocardiography demonstrated a reduced left ventricular ejection fraction (30%) with evidence of diastolic dysfunction. She was weaned off BiPAP after 24 hours, transitioned to nasal cannula oxygen, and discharged on a regimen including furosemide, metoprolol succinate, and lisinopril, with close cardiology follow-up.

## Discussion

The clinical presentation of this patient - acute dyspnea, elevated BNP, pulmonary congestion, and atrial flutter - was consistent with ADHF. BiPAP, as an NIV strategy, has demonstrated efficacy in managing acute cardiopulmonary decompensation without the risks associated with invasive mechanical ventilation [1,2]. It is particularly beneficial in patients with signs of respiratory distress or increased work of breathing, even in the absence of overt hypercapnia [3].

Atrial flutter with RVR in ADHF reflects a heightened sympathetic state and increased left atrial pressure due to elevated preload and afterload. The loss of effective atrial contraction impairs diastolic filling and further compromises cardiac output [4]. Early application of BiPAP can mitigate these effects by reducing intrathoracic pressure, lowering venous return (preload), and decreasing left ventricular afterload, thus improving stroke volume and oxygen delivery [5].

In this case, the initiation of BiPAP at IPAP 12 cm H₂O/EPAP 6 cm H₂O with FiO₂ 40% and a respiratory rate of 20 bpm resulted in rapid improvement in oxygenation, respiratory rate, and heart rate control, with spontaneous conversion from atrial flutter to sinus rhythm. This highlights the importance of hemodynamic unloading in rate control, especially in cases where atrial arrhythmias are secondary to pressure or volume overload. Several studies support the concept that afterload and preload reduction in heart failure patients can restore autonomic balance, lower circulating catecholamines, and facilitate rhythm stabilization [6,7]. Aggressive management of congestion and hypertension can prevent or reverse atrial arrhythmias through improved atrial hemodynamics [8]. In addition, atrial flutter in patients with heart failure and reduced ejection fraction (HFrEF) has been shown to be independently associated with adverse outcomes unless rate or rhythm control is rapidly achieved through optimization of volume status and medical therapy [9].

Another key clinical improvement in this case was the avoidance of endotracheal intubation and mechanical ventilation. The patient presented with severe respiratory distress and hypoxemia (SpO₂ 89% on room air) but responded quickly to NIV. This outcome is clinically meaningful, as mechanical ventilation in patients with ADHF is associated with increased morbidity, including ventilator-associated pneumonia, prolonged ICU stay, and delirium [10]. The ability to stabilize this patient with BiPAP and medical therapy alone represents an example of evidence-based, resource-conscious critical care management.

## Conclusions

This case highlights the effective use of early BiPAP in the management of ADHF presenting with respiratory distress and atrial flutter. The intervention resulted in rapid respiratory and hemodynamic improvement, spontaneous rhythm conversion, and avoidance of intubation, all of which underscore the clinical importance of prompt non-invasive support. BiPAP should be considered a key component of guideline-based therapy in ADHF, particularly when paired with vasodilators and diuretics. While this is a single-patient report, the findings support existing literature and underscore the importance of early intervention.

This case also aligns with existing evidence showing that atrial flutter in patients with HFrEF is associated with poor clinical outcomes unless treated early with rate or rhythm control, further reinforcing the importance of hemodynamic stabilization as an essential therapeutic target. Further large-scale studies are needed to determine optimal patient selection, timing, and ventilatory parameters. Lastly, health equity must be addressed - disparities in access to advanced therapies like BiPAP continue to affect outcomes in resource-limited populations. Expanding equitable access to NIV is essential to improving morbidity and mortality in patients with heart failure.
